# Development of a Mobile Health App (TOGETHERCare) to Reduce Cancer Care Partner Burden: Product Design Study

**DOI:** 10.2196/22608

**Published:** 2021-08-13

**Authors:** Ingrid Oakley-Girvan, Sharon Watkins Davis, Allison Kurian, Lisa G Rosas, Jena Daniels, Oxana Gronskaya Palesh, Rachel J Mesia, Arif H Kamal, Michelle Longmire, Vasu Divi

**Affiliations:** 1 Medable Inc Palo Alto, CA United States; 2 Stanford University Stanford, CA United States; 3 Duke University Durham, NC United States

**Keywords:** cancer, oncology, mHealth, caregiver, cancer survivor, mobile app, smartphone, feasibility, caregiver burden, symptom reporting

## Abstract

**Background:**

Approximately 6.1 million adults in the United States serve as care partners for cancer survivors. Studies have demonstrated that engaging cancer survivors and their care partners through technology-enabled structured symptom collection has several benefits. Given the high utilization of mobile technologies, even among underserved populations and in low resource areas, mobile apps may provide a meaningful access point for all stakeholders for symptom management.

**Objective:**

We aimed to develop a mobile app incorporating user preferences to enable cancer survivors’ care partners to monitor the survivors’ health and to provide care partner resources.

**Methods:**

An iterative information gathering process was conducted that included (1) discussions with 138 stakeholders to identify challenges and gaps in survivor home care; (2) semistructured interviews with clinicians (n=3), cancer survivors (n=3), and care partners (n=3) to identify specific needs; and (3) a 28-day feasibility field test with seven care partners.

**Results:**

Health professionals noted the importance of identifying early symptoms of adverse events. Survivors requested modules on medication, diet, self-care, reminders, and a version in Spanish. Care partners preferred to focus primarily on the patient’s health and not their own. The app was developed incorporating quality-of-life surveys and symptom reporting, as well as resources on home survivor care. Early user testing demonstrated ease of use and app feasibility.

**Conclusions:**

TOGETHERCare, a novel mobile app, was developed with user input to track the care partner’s health and report on survivor symptoms during home care. The following two clinical benefits emerged: (1) reduced anxiety among care partners who use the app and (2) the potential for identifying survivor symptoms noted by the care partner, which might prevent adverse events.

**Trial Registration:**

ClinicalTrials.gov NCT04018677; https://clinicaltrials.gov/ct2/show/NCT04018677

## Introduction

Approximately 6.1 million adults in the United States serve as care partners for cancer survivors [1]. Informal care partners, defined as unpaid spouses, relatives, and friends of the survivor, are essential partners with oncology teams in the delivery of complex cancer care services at home [2]. Care partners assist with activities of daily living, medication administration, wound care, transportation, meals, finances, advocacy, and emotional support. Care partners frequently attend medical visits with the survivor, often keeping track of physician instructions and medication changes [3]. Based on data collected through the 2015-2017 Behavioral Risk Factor Surveillance System (BRFSS) run by the Centers for Disease Control and Prevention (CDC), 24% of adults aged 45 years or older were care partners for relatives or friends [4]. Approximately 25% of those caring for cancer survivors spend more than 40 hours a week providing these services to their family or friends [5].

Studies have demonstrated that engaging cancer survivors and their care partners through technology-enabled structured symptom collection has several benefits [6-9]. For example, in a randomized trial, Basch et al found an increase in health-related quality of life and a decrease in emergency room visits and hospitalizations for survivors who were provided with a tablet computer–based symptom reporting system [10]. Studies have also found that survivor symptom self-reporting (patient-reported outcomes [PROs]) resulted in an increase in survival compared with usual care [8,11-13].

Complementary to patient reports, care partners bring a different and important perspective (observer-reported outcomes [ObsROs]) when reporting on survivor symptoms and may notice symptoms the survivor does not. Further, data demonstrate that these perspectives are feasible to collect. One study demonstrated that a series of systematic questions presented to care partners of children in palliative care were easy to complete and identified symptoms underdiagnosed by medical teams. Reporting by children aged 7 years or over and their care partners were consistent for common symptoms, but care partners reported irritability and nervousness more frequently than children [14]. In adult prostate cancer survivors, survivors who had a care partner were more likely to discuss pain at doctor visits than those without a care partner. This study concluded that tools encouraging early symptom reporting could lead to enhanced symptom and disease management [15]. Care partners’ assessments of symptoms in survivors were similar with survivor self-reports, indicating that the care partner could serve as a proxy [16,17]. There is also evidence that using an electronic symptom reporting system decreases the emotional distress of care partners [18,19].

Given the high utilization of mobile technologies, even among underserved populations and in low resource areas [20-24], mobile apps may provide a meaningful access point for all stakeholders for symptom management [25-27]. A Deloitte survey of US health care consumers found the following three key areas of consumer engagement: (1) consumers want to partner with clinical teams on their health care and management; (2) consumers are increasingly trusting and using online information; and (3) consumers, particularly those with chronic conditions, are increasingly utilizing health technologies [28], providing additional reasons to consider this avenue of engagement. Mobile health technologies show promise as solutions for health care needs across the cancer continuum [29] and have the potential to improve health care outcomes by providing consumers with a platform that can address all three of these key areas.

The development of most mHealth apps does not involve user input [30], despite evidence that incorporating feedback from appropriate stakeholders, including care partners, into smartphone app development can result in a more successful mobile tool [31,32]. The purpose of this study was to design and develop a mobile app in collaboration with users and other stakeholders for informal care partners to remotely monitor cancer survivors’ health and for providing care partner resources. While additional work is required to confirm the clinical effectiveness for specific outcomes, this paper documents the development process of this app (TOGETHERCare) and the preliminary results of early user testing.

## Methods

### Overview

This study was conducted in three sequential phases guided by the Technology Acceptance Model and user-centered design principles. The objective of phase 1 was to understand current care gaps and needs in cancer care through interviews with health care providers, survivors, and care partners. In phase 2, we gathered design and content specifications for the planned app through semistructured interviews with care partners, survivors, and clinicians. Finally, in phase 3, we created the first version of the TOGETHERCare mobile app for informal care partners to remotely monitor cancer survivors’ health and their own health and for providing care partner resources. We collected feedback from a small beta testing group of care partners who used the app for 28 days in two geographically different academic cancer centers (Duke and Stanford). This study has been registered on clinicaltrials.gov (NCT04018677). This iterative information gathering process was conducted to address three main questions. Table 1 lists the questions and our methods for addressing the questions.

**Table 1:** Main questions and methods for addressing the questions.

Main questions	Methods to address the questions
Phase 1. What are the needs and gaps for cancer care partners?	Discussions with 138 stakeholders including 32 care partners
Phase 2. What features would users like to see in such an app?	Semistructured interviews with three physicians, three cancer survivors, and three care partners
Phase 3. Is an mHealth^a^ app for cancer care partners feasible for them to use?	28-day beta iOS (Apple) user testing with feasibility and acceptability feedback from seven care partners in two geographically diverse academic cancer centers (Duke University and Stanford University)

^a^mHealth: mobile health.

### Phase 1. Stakeholder Discussions

We talked to stakeholders who would have insights into the needs of cancer patients, including providers, pharmaceutical company scientists, advocates, social workers, medical directors, researchers, and care partners. Stakeholders were identified first through Project Team Advisory Group (PTAG) members (N=14) and Patient Advocacy Council (PAC) members (N=4). PTAG members included survivor advocates, clinicians, and researchers with direct experience working with cancer survivors. PAC members included cancer survivors and care partners. A snowball technique was used to identify additional stakeholders, so that each informant identified additional people to interview until saturation of concepts was achieved.

The purpose of the interviews was to identify gaps in or barriers to cancer care, care partners, workflows, and the potential value of possible app components to organizations and individuals. These interviews were open-ended and did not use interview guides. Responses to the interviews were categorized by the research team [33].

### Phase 2. Semistructured Interviews

From a convenience sample from the Stanford Cancer Institute and Monterey-Salinas California health care systems, two interviewers conducted nine interviews using Institutional Review Board (IRB)-approved semistructured guides. The interviews were conducted with three physicians who work with cancer survivors, three cancer survivors, and three care partners currently caring for cancer survivors. Three interviews with Spanish-speaking survivors and care partners were conducted with a medical interpreter translating for the interviewer. Notes from the interviews were transcribed into a prepared template. The results were compiled, and response concepts were coded by two members of the core team.

### Phase 3. Beta Test

We conducted a beta test with cancer care partners to assess feasibility. Informed consent was collected through the app. The app included the following tabs: “Profile” for care partner’s name and demographics; “Activities” for informed consent, Health Insurance Portability and Accountability Act authorization, PRO surveys related to care partners’ health, survivor demographics, and surveys related to survivors’ health, including a targeted symptom list and a preappointment concerns survey; and “Resources” with links to resources related to care partners’ health, caregiving tasks, and local referral information.

The aim of the beta test was to test the consent and enrollment process and gather feedback from real care partners. Beta testing was also used to catch software or interoperability bugs before extensive usability testing. Cancer survivors (n=6) were recruited by staff at Stanford and Duke Universities and asked to consent to the project and identify their care partners. After providing informed consent, the care partners (n=7) (one survivor named two care partners) were enrolled in the study and instructed on how to download the app and enter data. Stanford University recruited survivors from survivor support groups, and Duke University recruited survivors from the palliative care clinic.

We conducted semistructured interviews with most beta participants at day 7 and all participants at day 28 to see what they liked and did not like about using the app. Interviews were zoom calls, typically 30 to 45 min in length, and the same script was followed in each interview, except for the first question (“Tell us about your care partner situation”) that we did not ask at the day 28 interview. Interviews provided much more in-depth information about care partners’ experiences using the app than we would have obtained using generic app satisfaction scales such as the System Usability Scale (SUS) [34,35] and mHealth App Usability Questionnaire (MAUQ) [36]. Interviews were recorded and transcribed.

Transcripts of the interviews were coded to identify themes. The thematic coding was done based on the principles of content analysis, where textual data are identified, analyzed, and grouped to form meaningful categories [37]. The level of analysis was entire sentences, as the interviews were semistructured and coding by word would give undue weight to a single respondent who had longer responses. Sentences from the interviews were coded based on the main concept contained in them, and the most commonly occurring themes across interviews were compiled.

Statistical methods for analyzing the in-app surveys included frequency counts and percentages to determine the completion rate of each survey, the number of surveys that were completed during the 4-week beta test, and the time required to complete each survey.

All procedures and study materials were independently approved by IRBs at Stanford and Duke Universities (clinicaltrials.gov NCT04018677). Because this was an unrandomized trial, no CONSORT checklist was filed. All procedures were in accordance with the ethical standards of the institutional and/or national research committee and with the 1964 Helsinki Declaration and its later amendments or comparable ethical standards. Informed consent was obtained from all participants included in the study.

## Results

### Phase 1. Stakeholder Discussions

The responses from the 138 stakeholders resulted in 262 responses that fell into different categories as follows: burnout/stress (n=108), cancer types/comorbid conditions (n=65), revenue/business (n=40), office value (n=22), insurance (n=12), and media/events attended by respondents (n=15). The major categories that emerged across all areas included the following: (1) survivors who come in for crisis visits are older, have comorbidities, have care partners at home who are burned out, live geographically far away, or have difficulty accessing appropriate resources and services; (2) buyers of new applications are risk sensitive or want a tool supported by validation studies proving reduction of clinical burdens and crisis visits when care partners are supported and less stressed; and (3) fitting into the electronic health record (EHR) system is important.

Responses by the 108 stakeholders who commented on burnout/stress are detailed in Table 2. Scheduling, keeping upcoming visits organized, or feeling overwhelmed with too much information related to different doctors, medications, and appointments were mentioned 27 times by most stakeholders across job functions.

The 32 care partners who were interviewed wanted an app to help them (1) be better organized and feel prepared to take care of their loved ones, including organizational support for scheduling visits, keeping up on the treatment and following treatment protocols, or knowing what signs/symptoms to look out for in order to identify adverse events earlier; (2) be more knowledgeable about various side effects, insurance options, treatment options, and other resources available to the survivor and care partner; and (3) have a sense of support and understanding of challenges from the clinical team, friends, family, and coworkers.

**Table 2:** Stakeholder discussion responses related to burnout and stress.

Stakeholder	Primary cause of stress/burnout for informants						
	Confusion navigating the health care system/insurance	Schedule/organization/too much info (meds, doctors, and appointments)	Socioeconomic costs, missing work	Distance from the doctor’s office/transportation	Elderly survivor	Emotional burden/depression	Other
Clinician/pharma (n=16)	2	3	3	3	1	3	1
Researcher (n=3)	0	1	0	1	0	0	1
Care partner (n=9)	1	4	2	0	0	1	1
Social worker/counselor (n=23)	3	7	3	4	1	3	2
Nurse (n=9)	2	3	2	1	1	0	0
Attorney (n=2)	1	1	0	0	0	0	0
Chief medical officer (n=1)	0	0	0	0	0	1	0
Clinician (n=17)	0	3	4	3	3	4	0
Patient advocate (n=4)	0	0	2	1	1	0	0
Chief executive officer (n=7)	0	2	1	1	1	1	1
Medical doctor (n=17)	2	3	3	2	2	4	1
Total (n=108)^a^	11	27	20	16	10	17	7

^a^108 of the 132 stakeholder comments were classified into the burnout/stress category.

### Phase 2. Semistructured Interviews

The demographics for the semistructured interviewees were quite varied. Physicians (n=3) were between 39 and 66 years of age and had been providing health care for those aged 14 to 39 years. There were two medical oncologists and one surgeon. Survivors (n=3) ranged in age from 43 to 74 years and had been diagnosed between 8 months and 19 years prior. Cancer diagnoses included breast cancer, melanoma, ovarian cancer, colon cancer, and thyroid cancer (one survivor had multiple diagnoses). Care partners (n=3) ranged in age from 22 to 63 years and had been care partners for a range of less than 1 year to 6 years. We interviewed two white non-Hispanic females, one white non-Hispanic male, one Asian male, three Latino females, and two Latino males.

For many of the coded interview concepts, there was general agreement across the physicians, cancer survivors, and care partners. Although all three groups agreed that there is currently no systematic way for specialists to keep in touch with survivors once they have moved to community care, survivorship care plans (SCPs) would be useful. The SCP provides treatment history, management of side effects, and information on who to contact. However, they currently do not receive or prepare an SCP. All three groups concurred that the survivor had to initiate either a visit or call to the specialist. All three groups agreed that they have smartphones and that an app including the ability to communicate between the different groups, along with other modules, such as guidance on assisting with daily medical tasks and activities of daily living, would be useful.

There were also differences between the three groups of semistructured interviewees in concepts coded for responses to four of the interview questions. Care partners and survivors had different kinds of questions they would like to ask physicians, compared with questions clinical staff felt they heard frequently from care partners. Cancer survivors indicated no concerns about the app, and one care partner mentioned a concern about keeping medical information private, but clinical staff were concerned about the added workload and whether the app would prove useful unless all members of the care team participated in the effort. Clinical staff had specific ideas for smartphone app modules, including communication tips for survivors and care partners to make better use of their time with the clinical team; a dashboard or status bar that would track the survivor through the care process, from the initial treatment through all treatments provided by different specialties; and addition of PROs or ObsROs. Survivors and care partners were interested in modules on medication, diet, self-care, reminders, and a version in Spanish.

### Phase 3. Beta Test Qualitative Analysis

Evidence from the beta test semistructured qualitative surveys indicated that care partners (n=7) found the app to be useful, and would continue to use it, as well as recommend it to others. They also suggested that adding features to the app, such as the ability to search for specialized information and insert open-ended notes, would greatly enhance the functionality of the app. Care partners were focused on survivor health, and were not too interested in responding to health questions about themselves; instead, they felt that most of the questions should focus on the survivor. Moreover, they requested more feedback from the daily surveys, such as an explanation of what their survivors’ health measurements indicated in terms of the survivors’ current health statuses. The four main themes that emerged from the beta test qualitative interviews are shown in Table 3.

**Table 3:** Beta test qualitative analysis themes with illustrative comments.

Theme	Concept	Selected comments
The app is useful	All care partners found the app to be useful in multiple ways, including reducing their anxiety by focusing on the fixed number of survey questions, as well as serving as a learning tool and raising their level of awareness.	*Finds it very helpful. Helps her not to worry and eases her mind. Helps keep things in perspective - she focuses on set number of questions, not a huge list.* *I thought the measurement of mental health was helpful. It might help avoid depression. Well, I think it's easy to use and to understand.*
Add functionality to the app	Care partners suggested that the app should provide specialized information and contain an open-ended notes section.	*Especially if more types of information could be added to this app that I can use other than general Information about caregiving.* *Would be nice to have a box where you can put notes in - especially about the survivor.*
Care partners are focused on survivor health	Care partners remarked that the surveys contained too many questions pertaining to the care partners rather than focusing on survivors.	*I want to focus more on the cancer patient because that person is the one who needs help, more than the care partner.* *Actually, I would rather focus more on the cancer patient myself.*
Care partners need more feedback	Care partners noted that they would have liked feedback about their survey responses.	*It would have helped me to know that someone on the end is monitoring my responses or I could receive a response back when I did things on the app.* *It would be better if you could receive some kind of report back of what the measurement means, especially about patient’s health and stress level, depression, exercise.*

### Phase 3. Beta Test Quantitative Data Analysis

Seven care partners participated in the beta test. The completion rates below refer to the percentage of surveys for which care partners answered all the questions. During the beta test, care partners were told that the demographics section was optional, so that was the least frequently completed survey (Stanford, 25%; Duke, 33%). The Patient Health Questionnaire-4 [38] survey about the care partner, which measures depression and anxiety in four items, was completed only 31% of the time by both groups combined, with the care partners infrequently answering any of the questions. Demographics about the survivor were completed 90% of the time, and “My loved one’s health” (about the survivor) and the preappointment survey were completed 91% of the time. All other surveys were completed between 97% and 100% of the time by both groups combined.

Within the 4-week test period, care partners were expected to answer each survey a certain number of times. Table 4 lists the survey name, the frequency with which the survey came up on the app, the number of times it was available during the 28-day beta test time frame, and the median and mean numbers of care partner beta testers (n=7) who started the survey. Care partners started surveys close to the frequency at which they were available, but not all the survey questions were completed (participants could skip any questions they did not wish to answer). The “number started” refers to the number of surveys for which at least one question was answered, regardless of whether all questions in that survey were completed. One-time surveys took on average from 32 seconds (care partner eligibility) to 211 seconds (e-consent document and signature [consent previously explained and reviewed by the clinical team]) to complete. Repeated surveys were completed, on average, in 2 minutes or less. The Patient Health Questionnaire-4 (PHQ-4) had an average completion time of 8 seconds, and the “My loved one’s health” was completed on average in 129 seconds.

**Table 4:** Surveys included in the care partner app by availability frequency and mean number started by beta testers (n=7) during the beta testing period (all surveys were completed by the care partner).

Survey name	Requested Frequency	Number of times available	Median number started	Mean number started
Care partner consent	Once	1	1	1.29
Care partner eligibility	Once	1	1	1.14
Daily (about care partner sleep and mood)	Daily	25	9	13.86
Demographics (about care partner)	Once	1	1	1.00
EQ5D^a^ [39] (about care partner)	Weekly	5	5	4.86
My loved one (survivor demographics)	Once	1	1	1.14
My loved one’s health (about survivor)	Weekly	5	5	4.57
PROMIS^b^ [40] (about care partner)	Biweekly	4	3	2.86
PHQ-4^c^ [38] (about care partner)	Biweekly	4	3	2.71
Preappointment (care partner concerns re: survivor appointment)	Weekly	5	5	4.71
Weekly (about care partner health)	Weekly	5	5	4.86

^a^EQ5D: EuroQol 5-Dimension.

^b^PROMIS: Patient-Reported Outcomes Measurement Information System.

^c^PHQ-4: Patient Health Questionnaire-4.

## Discussion

This paper describes the user-involved development process of TOGETHERCare, a smartphone app for care partners. This is one of few studies regarding app development for cancer care partners that utilized a rigorous development approach involving users [31,41]. Engaging users in the design of an mHealth app facilitates app adoption and usage [42].

As the US population ages, more care is being delivered at home. Limits on rehabilitation and nursing home payments can result in survivors being discharged before they are ready [43,44]. Starting in 2015, more money was spent nationally on home care than care provided in nursing homes [45]. This increase in caring for survivors at home has raised the burden on informal care partners, such as family and friends. This heavy burden can affect mental and physical health, and the care partner’s health can appreciably impact the survivor [2,46-51]. Clinical benefits have been associated with PROs [10-13]. While studies on care partner reporting are limited [19], there is evidence that care partners can identify early symptoms [14-16], and the use of a symptom reporting system may reduce caregiver distress [18].

TOGETHERCare is a mobile app that provides for care partner symptom reporting for themselves and the survivors. The following three phases were completed in the development of the app: (1) stakeholder discussions, (2) semistructured interviews, and (3) beta testing of the app. These phases are essential to ensure that the app is developed incorporating user preferences to increase its value [30].

In our stakeholder discussions, all stakeholders felt that an app for informal care partners would be beneficial. Stakeholders frequently mentioned that care partners feel overwhelmed with too much information and financial considerations, and have emotional issues including stress and depression. Clinical staff, care partners, and survivors included in the semistructured interviews all agreed that an app designed to help care partners would be welcome. Results of early user beta testing showed that TOGETHERCare is feasible to use, with users able to complete surveys and commenting that the app was useful and helpful. Our qualitative interviews with testers revealed that care partners are primarily focused on their survivor’s health, not their own, and the quantitative analysis indicated the need to reduce the number of surveys about care partner’s health. Several care partners mentioned that the survivor-focused surveys helped to reduce their anxiety and bring relief by reducing the universe of things they had to worry about. Similar findings were observed by Chih et al, who examined an online symptom-reporting system for advanced cancer survivors [18].

This study has some limitations. While an open-ended interview with stakeholders allowed us to receive perspectives that might otherwise not have been discussed, having a more structured interview may have allowed us to examine opinions in a more standardized way. Limitations in this study also include the small convenience sample of care partners involved in the semistructured interviews and the beta testing. However, small sample sizes for exploratory studies are common, and other published research studies on app development and acceptability have used between 5 and 11 users for feasibility and usability testing [52-54]. The beta test version of the app did not include visualizations that are expected to be developed in future versions of the app, although mockups of visualizations were presented during the qualitative interviews, and feedback was obtained. Survey data completed by care partners were not presented within the EHR to clinicians because we were not testing the feedback component at this stage. Future work in a larger clinical sample will include providing survey data completed by care partners to clinicians within a work flow they are already using. Care partners in our study were required to have an iPhone, as the beta test version was developed for the iOS platform, with a future intention to include Android phones.

Next steps include testing the app with a larger population, providing data recorded by care partners about their survivors’ symptoms to the clinical team within the EHR, and ultimately testing the app impact on specific outcomes in a randomized controlled trial. Further evaluation in a randomized clinical trial is needed to provide evidence that the app would result in fewer hospitalizations and emergency room visits and potentially extend survival.

TOGETHERCare, a novel mobile app, was developed together with care partners, health care professionals, and survivors to track the health of care partners and report on a targeted list of survivor symptoms during home care. The following two clinical benefits have emerged: (1) the reduction of anxiety among care partners who use the app and (2) the potential for the identification of symptoms exhibited by the survivor and noted by the care partner, which, if caught early, might prevent unnecessary emergency room or hospital care.

## Abbreviations

EHR: electronic health record
IRB: Institutional Review Board
ObsRO: observer-reported outcome
PAC: Patient Advocacy Council
PRO: patient-reported outcome
PTAG: Project Team Advisory Group
SCP: survivorship care plan
